# Estimated dietary intake and major food sources of polyphenols in the Polish arm of the HAPIEE study^☆^

**DOI:** 10.1016/j.nut.2014.04.012

**Published:** 2014-11

**Authors:** Giuseppe Grosso, Urszula Stepaniak, Roman Topor-Mądry, Krystyna Szafraniec, Andrzej Pająk

**Affiliations:** a Department of Clinical and Molecular Biomedicine, Section of Pharmacology and Biochemistry, University of Catania, Catania, Italy; b Department of Epidemiology and Population Studies, Jagiellonian University Medical College, Krakow, Poland

**Keywords:** Polyphenols, Intake, General population, Food sources, Polish adults

## Abstract

**Objective:**

The aim of this study was to estimate the intake of known individual polyphenols and their major dietary sources in the Polish arm of the HAPIEE (Health, Alcohol and Psychosocial factors In Eastern Europe) study.

**Methods:**

A total of 10,477 random sample (45–69 y) of urban population of Krakow, Poland, completed a validated 148-item food frequency questionnaire. Polyphenol intake was calculated by matching food consumption data with the recently developed Phenol-Explorer database.

**Results:**

The mean intake of polyphenols was 1756.5 ± 695.8 mg/d (median = 1662.5 mg/d). The main polyphenol groups were flavonoids (897 mg/d) and phenolic acids (800 mg/d). A total of 347 polyphenols from 19 polyphenol subclasses were found. The individual compounds with the highest intakes were isomers of chlorogenic acid (i.e., 5-caffeoylquinic acid and 4-caffeoylquinic acid) among hydroxycinnamic acids (average intake 150 mg/d), that largely originated from coffee, and compounds belonging to the catechin chemical family (i.e., [+]-gallocatechin, [-]-epigallocatechin 3-O-gallate, and [-]-epicatechin) among flavanols (average intake 50 mg/d), that mostly originated from tea and cocoa products.

**Conclusions:**

The current study provides the most updated data for individual polyphenols intake in the diet of a well-established nutritional cohort. These findings will be useful to assess potential beneficial role on health of specific foods with high polyphenol content and characterize the effects of individual phenolic compounds.

## Introduction

Fruit and vegetable consumption has been shown to provide a protective effect against cardiovascular disease (CVD) [1], [2], [3], [4]. In the past 10 y, a rise in interest also on tea, cocoa, coffee and wine consumption has been motivated by their content of polyphenols [5], [6]. These compounds constitute a heterogeneous group of molecules divided into five main classes according to their chemical structure: flavonoids, phenolic acids, stilbenes, lignans, and others [7]. The considerable diversity of their structures makes polyphenols different from other antioxidants. Their bioavailability and biologic properties vary to a great extent and are affected by their chemical structure [8]. Furthermore, the conjugation reactions with methyl, sulfate, or glucuronide groups and the nature and amounts of metabolites formed by the gut microflora, may influence their absorption and their bioavailability [8], [9], [10], [11].

Despite the promising evidence regarding the possible role of polyphenols in disease prevention, data regarding their consumption at the population level is not strong enough to suggest optimal intake levels and dietary recommendations [12]. Some recent European investigations pointed out the importance of using a similar methodology to allow comparisons and to reduce the variability of estimated dietary intake of compounds among studies [13], [14]. The most recently used tool was the Phenol-Explorer database (www.phenol-explorer.eu), a comprehensive database containing food composition data of 502 polyphenols in 452 foods [15]. A reasonable comparison of polyphenol intake is possible due to a similar methodology reported in both the French SU.VI.MAX (Supplementation en Vitamines et Mineraux Antioxydants) and the Spanish PREDIMED (Effects of Mediterranean Diet on the Primary Prevention of Cardiovascular Disease) cohorts [13], [14]. However, despite the emerging interest regarding polyphenol consumption among the population, information on Central and Eastern European countries is poor. A Polish study assessed the total polyphenol intake of the population investigated [16], however, the analysis did not include information regarding specific polyphenol classes and individual polyphenols. The aim of this study was to estimate the dietary polyphenol intake in a well-established nutritional cohort, describe the intake of individual polyphenols, and document the contribution of specific foods to total and individual polyphenol intake.

## Materials and methods

### Study population

The study group constituted of participants in the Polish arm of the HAPIEE (Health, Alcohol and Psychosocial factors In Eastern Europe) study, a prospective cohort study aimed to investigate the determinants of CVDs and other chronic conditions in Central and Eastern Europe. The study protocol, rationale, design, and methods have been described in detail elsewhere [17]. Briefly, 10 728 adults (ages 45–69 y) from Krakow were randomly selected at the baseline survey conducted in 2002–2005 (response ratio, 59%). The participants provided written informed consent and the study protocol was approved by the ethics committee at University College London, United Kingdom and by the bioethics committee of the Jagiellonian University (no. KE/99/03/B/284 2).

Data regarding demographics (i.e., age, sex, educational and occupational levels) and lifestyle characteristics (i.e., physical activity, smoking and drinking habits) were collected. Educational level was categorized as low (primary/secondary), medium (high school), and high (university). Occupational level was categorized as low (unskilled/unemployed workers), medium (partially skilled workers), and high (skilled workers). Physical activity level was categorized as daily low active (expended energy <16.7 kJ [<4 kcal]/min), moderately active (expended energy 16.7–29.3 kJ [4–7 kcal]/min), and highly active (expended energy >29.3 kJ [7 kcal]/min). Smoking status was categorized as nonsmoker and current smoker. Alcohol consumption was categorized as none or moderate drinker (<12 g/d) and alcohol drinker (>12 g/d).

### Dietary assessment

Dietary data were collected using a food frequency questionnaire (FFQ) based on a previously developed tool [18] and subsequently adapted in the Whitehall II Study [19]. The FFQs consisted of 148 food and drink items representative of the diet during the preceding 3 mo. A country-specific instruction manual that included photographs to facilitate the estimation of portion sizes was used. Participants were asked how often, on average, they had consumed that amount of the item during the past 3 mo, with nine responses ranging from *never or less than once per month* to *six or more times per day*. Moreover, participants were asked to include additional foods and frequency of consumption by manual entry. We excluded 251 questionnaires with extreme energy intakes (<500/>4000 kcal/d for women and <800/>5000 kcal/d for men).

### Estimation of polyphenol intake

Data on the polyphenol content in foods were obtained from the Phenol-Explorer database (www.phenol-explorer.eu) [15]. Foods that did not contain polyphenols were excluded from the FFQ. Items on the FFQ containing more food components were separated according to their ingredients and weight loss or gain during cooking was corrected using yield factors [20]. Several products that contained refined wheat flour were included in the list of foods (i.e., pizza, biscuits) and polyphenol content in these foods was estimated from their wheat flour contents. The average food consumption was calculated (in g or mL) by following the standard portion sizes used in the study and then converted in 24-h intake. Finally, an advanced search was carried out in the Phenol-Explorer database to retrieve mean content values for all polyphenols contained in the foods obtained and individual polyphenol intake from each food was calculated by multiplying the content of each polyphenol by the daily consumption of each food. Accordingly, the database also allows calculating the aglycones. Total polyphenol intake was calculated as the sum of all individual polyphenol intakes from all food sources encountered according to this process. As described in previous studies with similar methodology [13], [14], the polyphenol content of foods included in the FFQ that could corresponded to several entries in the Phenol-Explorer database (i.e., “wine,” “fruit juice,” “nuts,” and “seeds” could have corresponded to a number of foods in Phenol-Explorer database) was weighted according to their consumptions in the Polish population.

### Statistical analysis

Data are presented as medians, means, and SDs for continuous variables and frequencies and percentages for categorical variables. Total polyphenols in the Polish HAPIEE cohort were determined for the whole study sample as well as according to the sociodemographic and lifestyle characteristics. Polyphenol intake distribution was controlled by Kolmogorov-Smirnov test and it roughly followed a normal distribution, partially asymmetric due to extreme values on the upper side. Thus, we also reported median values to compare differences in intakes between groups by using Mann-Whitney *U-*test and Kruskall-Wallis test, as appropriate. Polyphenols may be associated with total energy intake either because they contribute directly to energy intake or because individuals who consume more total energy also eat, on average, more of all specific nutrients, thus polyphenol intake was also calculated in energy-adjusted terms (mg of nutrient per 1000 kcal/d of total energy consumed).

Mean intakes of all individual polyphenols, polyphenol groups (phenolic acids, anthocyanidins, flavones, flavonols, flavanones, proanthocyanidins, ellagitannins, isoflavonoids, and lignans) and major food contributors were determined. All reported *P*-values were based on two-sided tests and compared with a significance level of 5%. SPSS 17 (SPSS Inc., Chicago, IL, USA) software was used for all the statistical calculations.

## Results

### Total polyphenols intake

A total of 10 477 participants were available for the final analyses. Among 148 food items considered in the HAPIEE questionnaire, 80 contained relevant polyphenols according to the Phenol-Explorer database. In all, 347 polyphenols from 19 polyphenol subclasses were found in these foods. The mean intake of polyphenols was 1756.5 ± 695.8 mg/d (median = 1662.5 mg/d). These values included intakes of sugars and polyols linked to aglycones, thus the total intake of aglycones was determined, resulting in 1192.3 ± 412 mg/d (median = 912 mg/d). Nutrient density by energy intake was also calculated, with a mean intake of 854.3 ± 331.3 mg/d per 1000 kcal (Table 1).

**Table 1:** Total and energy-adjusted polyphenol intake According to demographic characteristics and distribution of Polish participants of the HAPIEE study (N = 10 477)

Characteristics	Polyphenol intake			*P*-value†	Energy-adjusted∗ polyphenol intake		*P*-value†
	N (%)	Median	Mean ± SD		Median	Mean ± SD	
Total population	10 477	1662.5	1740.7 ± 630.2		801.1	854.3 ± 331.3	
Sex				0.034			<0.001
Men	5137	1665.7	1755.4 ± 729.5		781.9	840.1 ± 317.9	
Women	5340	1659.1	1726.5 ± 661.5		817.1	869.6 ± 343.3	
Age class				<0.001			<0.001
<50 y	1933	1745.9	1817.1 ± 710.5		835.9	884.8 ± 353.3	
50–54 y	2161	1688.1	1763.5 ± 671.4		797.9	850.7 ± 313.7	
55–59 y	2194	1675.4	1758.7 ± 740.0		801.3	862.6 ± 367.4	
60–64 y	2084	1616.4	1693.3 ± 689.0		783.9	840.2 ± 315.5	
≥65 y	2105	1606.7	1675.4 ± 656.5		789.7	839.9 ± 301.9	
Educational level				<0.001			<0.001
Low	1210	1618.6	1688.1 ± 652.9		773.1	825.5 ± 309.2	
Medium	6269	1659.1	1729.5 ± 680.3		793.3	844.8 ± 326.4	
High	2987	1694.6	1787.1 ± 741.5		828.6	888.7 ± 347.7	
Occupational level				0.049			<0.001
Low	5550	1653.6	1727.8 ± 695.6		785.5	839.3 ± 317.8	
Medium	3499	1686.1	1764.7 ± 707.0		823.7	879.1 ± 354.7	
High	1126	1663.5	1744.1 ± 671.6		802.4	858.0 ± 317.8	
Physical activity				<0.001			0.041
Low	2944	1629.1	1710.3 ± 685.7		790.4	849.9 ± 336.6	
Medium	3585	1660.4	1738.0 ± 690.8		812.8	866.9 ± 333.2	
High	3365	1699.7	1776.8 ± 710.0		798.9	847.1 ± 324.6	
Smoking status				<0.001			0.033
Yes	3194	1697.6	1782.1 ± 709.5		809.3	866.2 ± 328.5	
Not	6700	1649.5	1724.3 ± 689.3		832.7	849.9 ± 332.7	
Alcohol drinking				0.017			<0.001
Yes	9477	1661.0	1739.0 ± 694.7		805.1	860.4 ± 332.6	
No	417	1780.9	1832.8 ± 727.6		701.9	736.6 ± 277.9	

∗ Per 1000 kcal.

† Evaluated by Mann-Whitney *U* test or Kruskall-Wallis test.

Polyphenol intake is presented according to demographic characteristics (Table 1). Intakes were slightly higher in men than in women (1771.2 ± 729.5 and 1742.3 ± 661.5 mg/d, respectively; *P* = 0.034), but when the energy-adjusted amount was calculated, women had a higher intake of polyphenols than men. Within the limited range tested, age had significant influence on total and energy-adjusted polyphenols intake, being higher among younger participants. Individuals with a university education level showed higher total and energy-adjusted polyphenol intake than those in lower educational categories, whereas different groups of occupational level had significantly different polyphenol intake only by energy intake. Increased means and medians of polyphenol intake were also found among those in the higher physical activity groups, smokers, and nondrinkers, but when comparing energy-adjusted polyphenol intake, drinkers had higher intake levels (860.4 ± 332.6 versus 736.6 ± 277.9 mg/d per 1000 kcal *P* < 0.001).

### Groups of polyphenol intake and main food contributors

The main polyphenol groups were flavonoids (897 mg/d, 572 mg/d as aglycone equivalents, 52% of total intake of polyphenols), phenolic acids (800 mg/d, 521 mg/d as aglycone equivalents, 46% of total intake of polyphenols), whereas alkylphenols (1.5%) and other polyphenols, such as lignans, stilbenes, and tyrosols accounted for lower proportions (1%) (Table 2). The mean daily intakes of flavanols (637 mg/d) in the flavonoids and hydroxycinnamic acids (705 mg/d) in the phenolic acids were high compared with others groups. Comparable mean intakes were found for flavonols, flavanones, and hydroxybenzoic acids (an average of 100 mg/d), whereas intake of anthocyanins, flavones, and dihydrocalcones was lower. When expressed as aglycone equivalents, the relative contribution of flavanols (412 mg/d) increased from about 30% to 35% and that of hydroxycinnamic acids (401 mg/d) was reduced from about 40% to 35%, whereas the relative contributions of the other polyphenol subclasses remained similar.

**Table 2:** Total, classes, and subclasses of polyphenol intake According to food group sources and main food contribution

Polyphenol class	Food group								Main food contributors (% contribution to polyphenol class)		
	Total foods	Nonalcoholic beverages	Alcoholic beverages	Fruits	Vegetables	Cereals	Seeds and oils	Others			
	mg/d per person (mean ± SD)										
Total polyphenols∗	1740.7 ± 630.2	1150.2 ± 501.3	9 ± 10.3	213.9 ± 112.2	88.8 ± 53.2	38.9 ± 29.8	72 ± 65.1	167.8 ± 112.3	Coffee (40)	Tea (27)	Chocolate (8)
Flavonoids†	897.6 ± 423.4	500.6 ± 131.3	6.4 ± 11.3	164.6 ± 106.5	46.9 ± 47.3	7.9 ± 8.2	13.5 ± 44.7	165.5 ± 294.9	Tea (48)	Chocolate (18)	Apples (8)
Flavanols	637.3 ± 311	388.8 ± 91.3	2.4 ± 12.5	69.6 ± 17.7	1.2 ± 15.5	–	13.5 ± 44.7	161.7 ± 290.5	Tea (60)	Chocolate (25)	Apples (7)
Flavonols	106.1 ± 89.2	51.1 ± 21.5	0.2 ± 1.5	15.2 ± 40.8	37.2 ± 33.5	–	<0.01	2.36 ± 4.2	Tea (47)	Onion (13)	Spinach (13)
Flavanones	103.8 ± 70.4	54.7 ± 64.9	2.9 ± 9	45.8 ± 58.2	0.3 ± 0.5	–	<0.01	–	Orange juice (29)	Squash (24)	Oranges (23)
Flavones	15.5 ± 11	5.2 ± 6.1	<0.1	0.7 ± 0.6	1.62 ± 1.81	7.93 ± 8.23	<0.01	–	Flour (51)	Orange juice (23)	Squash (10)
Anthocyanins	29.7 ± 93.3	<0.1	0.6 ± 3.5	22.7 ± 92.4	6.4 ± 5.4	–	–	–	Black currant (21)	Beans (19)	Strawberries (16)
Dihydrochalcones	11.3 ± 10.7	0.8 ± 1.1	–	10.6 ± 9.5	–	–	–	–	Apples (93)	Apple juice (7)	
Isoflavonoids	1.6 ± 0.2	–	<0.1	–	0.2 ± 0.2	–	–	1.4 ± 8.4	Soy meat (85)	Beans (12)	Soy milk (3)
Phenolic acids‡	800.2 ± 345.8	641.8 ± 403.6	2.1 ± 7.4	49.2 ± 6	41.8 ± 23.9	4.78 ± 4.9	58.15 ± 76.5	2.2 ± 4.1	Coffee (66)	Tea (12)	Vegetable oils (7)
Hydroxybenzoic acids	94.6 ± 72.9	85.4 ± 42.3	1 ± 3.6	6.8 ± 24.3	1.3 ± 2.6	0.10 ± 0.2	0.1 ± 1.7	–	Tea (89)	Apples (3)	Raspberries (2)
Hydroxycinnamic acids	705.5 ± 419.4	556.4 ± 307.2	1.1 ± 3.6	42.5 ± 5	40.4 ± 21.8	4.7 ± 1.7	58.1 ± 76.5	2.27 ± 4.07	Coffee (75)	Vegetable oil (8)	Apples (5)
Alkylphenols	26.1 ± 32.1	–	–	–	–	26.1 ± 32.1	–	–	Cereals (51)	Dark bread (45)	Pasta (3)
Alkylmethoxyphenols	3.7 ± 3.1	3.6 ± 3.1	–	–	–	–	<0.01	–	Coffee (98)	Beer (2)	
Lignans	0.6 ± 11.9	0.17 ± 0.1	<0.1	<0.1	<0.1	0.1 ± 0.1	0.3 ± 11.8	<0.01	Seeds (51)	Tea (27)	Dark bread (8)
Stilbenes	0.2 ± 0.6	–	0.1 ± 0.6	<0.1	<0.01	–	–	<0.01	Red wine (56)	Strawberries (14)	White wine (12)
Tyrosols	0.4 ± 3.3	–	0.3 ± 1.2	–	0.1 ± 3.2	–	<0.01	–	Beer (33)	Red wine (26)	Olives (20)

∗ Do not include hydroxybenzoketones, hydroxyphenilpropenes, methoxyphenols, naphtoquinones, furanocoumarins, hydroxycinnamaldehydes, hydroxycoumarins, and hydroxybenzaldehydes in traces.

† Do not include dihydroflavonols and chalcones in traces.

‡ Do not include hydroxyphenylpropanoic acids and hydroxyphenylacetic acid in traces.

The main dietary sources for the total polyphenols were coffee, tea, and chocolate, whereas fruits and vegetables accounted for a lower percentage of the total amount of polyphenols in the diet of the cohort (Table 2). Within each food group, the main beverage contributors were identified as coffee and tea for nonalcoholic and wine and beer for alcoholic beverages. For other food groups, chocolate and vegetable oils were the second most important contributors of total polyphenols intake. Among fruit, polyphenols were mostly coming from apples, oranges, and several berry fruits, whereas among vegetables, spinach and potatoes were the main contributors.

Coffee was the primary food item contributing to phenolic acid intake (66%) and also to the total intake of polyphenols (40%). Tea was identified as the most important source, especially for flavanols and flavonols, contributing almost 300 mg of daily flavonoid intake. Other important contributors to the intake of flavonols, flavanones, and flavones were fruits (especially apple and orange juices); flour products were the major source for flavones; black currant for anthocyanidins; apples for dihydrochalcones; and soy meat for isoflavones. Major contributors of intake of phenolic acids were coffee (75% of hydroxycinnamic acids), tea (89% of hydroxybenzoic acids), and vegetable oils (8% of hydroxycinnamic acids). Among other minor contributors, apples and raspberries contributed to 2% to 3% of total phenolic acids. Lignans were derived from seeds, tea, and bread, whereas stilbenes, tyrosols, hydroxycoumarins, and hydroxybenzaldehydes came mostly from alcoholic beverages (especially wine and beer).

### Intake of individual polyphenols

The intake of all individual polyphenols consumed in the Polish HAPIEE cohort was determined (see supplemental data online). Of the 347 consumed polyphenols, 108 had mean intake values >1 mg/d. The numbers of more commonly consumed polyphenols are shown in Table 3. The first 25 compounds on the list were all consumed in amounts >20 mg/d and all together accounted for 74% of the total polyphenol intake. As expected, most of them were phenolic acids and flavonoids, mostly among the hydroxycinnamic acids (seven compounds) and flavanols (14 compounds) classes. The individual compounds with the highest intakes were isomers of chlorogenic acid (i.e., 5-caffeoylquinic acid and 4-caffeoylquinic acid) among hydroxycinnamic acids (average intake 150 mg/d), that largely originated from coffee, and compounds belonging to the catechin chemical family, that is,(+)-gallocatechin, (-)-epigallocatechin 3-O-gallate, and (-)-epicatechin, among flavanols (average intake 50 mg/d) that mostly originated from tea and cocoa products (Table 3). The remaining compounds among these 25 polyphenols were two antocyanins (namely hesperidin and hesperetin) uniquely coming from citrus fruits (and juices).

**Table 3:** Intake of most consumed individual polyphenols and their main food sources

Polyphenol	Polyphenol subclass	Polyphenol intake, mg/day (mean ± SD)	Main food contributors (% contribution to polyphenol class)		
5-caffeoylquinic acid	Hydroxycinnamic acids	224.6 ± 112.7	Coffee (73)	Apples (11)	Potatoes (9)
4-caffeoylquinic acid	Hydroxycinnamic acids	149.1 ± 124.8	Coffee (94)	Tea (4)	Apples (1)
3-caffeoylquinic acid	Hydroxycinnamic acids	128.2 ± 111.6	Coffee (96)	Plums (2)	Tea (1)
(+)-gallocatechin	Flavanols	73.6 ± 64.8	Tea (100)		
5-O-galloylquinic acid	Hydroxybenzoic acids	60.8 ± 45.4	Tea (100)		
Polymers (>10 mers)	Flavanols	58.3 ± 43.8	Chocolate (68)	Nuts (15)	Strawberries (8)
(-)-epigallocatechin 3-O-gallate	Flavanols	48.1 ± 39.5	Tea (100)		
Procyanidin dimer B2	Flavanols	46.3 ± 34.7	Apples (61)	Tea (28)	Chocolate (7)
(-)-epicatechin	Flavanols	45.9 ± 34.2	Tea (45)	Apples (35)	Chocolate (14)
Ferulic acid	Hydroxycinnamic acids	43.9 ± 33.7	Coffee (68)	Flour (7)	Beans (6)
04–06 mers	Flavanols	43.4 ± 31.4	Chocolate (85)	Nuts (4)	Strawberries (4)
(-)-epicatechin 3-O-gallate	Flavanols	38.6 ± 22	Tea (100)		
(-)-epigallocatechin	Flavanols	38.0 ± 21.2	Tea (100)		
Hesperidin	Anthocyanins	37.5 ± 21.4	Squash (56)	Orange juice (44)	
Stigmastanol ferulate	Hydroxycinnamic acids	37.5 ± 22.6	Vegetable oils (100)		
Hesperetin	Anthocyanins	28.1 ± 12.3	Oranges (63)	Orange juice (20)	Lemons (15)
5-feruloylquinic acid	Hydroxycinnamic acids	27.9 ± 14.3	Coffee (99)	Carrots (1)	
03 mers	Flavanols	27.4 ± 12.2	Chocolate (93)	Plums (2)	Strawberries (2)
02 mers	Flavanols	26.7 ± 12.3	Chocolate (90)	Beans (3)	Plums (2)
Gallic acid	Hydroxybenzoic acids	25.0 ± 11.2	Tea (97)	Bananas (1)	Cauliflowers (1)
(+)-catechin 3-O-gallate	Flavanols	24.9 ± 12.3	Tea (100)		
Theaflavin 3′-O-gallate	Flavanols	21.4 ± 12.8	Tea (100)		
07–10 mers	Flavanols	20.5 ± 14.5	Chocolate (75)	Nuts (10)	Strawberries (7)
4-feruloylquinic acid	Hydroxycinnamic acids	20.4 ± 12	Coffee (100)		
Procyanidin dimer B1	Flavanols	20.0 ± 12	Tea (97)	Plums (1)	Apple juice (1)

## Discussion

To our knowledge, this study is the first to report a detailed description of the classes and individual polyphenol intake in a large nutritional cohort in Poland, and the largest among those studied. The mean polyphenol intake of this study (1756.5 mg/d; 1092.3 mg/d as aglycones) was higher than in the previous reports for the Polish population (1172 ± 354 mg/d for men and 1031 ± 320 mg/d for women) [16], as well as than in other studies reported in French (1193 ± 510 mg/d) [13], Spanish (820 ± 323 mg/d) [14], and Finnish (863 ± 415 mg/d) [21] populations. Flavonoids intake (897 mg/d) was higher than those reported for United States [22], Denmark [23], Finland [24], [25], the Netherlands [26], and Japan [27] and similar to that reported for tea consumers in the U.S. population [28]. Smaller differences were seen regarding the intake of phenolic acids (800 mg/d) compared with those reported in French [13], Spanish [14], and Finnish populations [21]. Flavanols (proanthocyanidins) were the second most abundant flavonoid consumed in all cohorts, whereas other polyphenol classes were consumed less, accounting for about 1 mg/d per person, due to their low contents in foods (lignans and stilbenes) or to the low consumption of their main dietary sources (isoflavones).

The aforementioned differences in polyphenol intake may depend on country-specific food preferences and, consequently, on preferences for main dietary sources. Indeed, the main food contributors to polyphenol intake were mostly represented by nonalcoholic beverages (such as tea and coffee) that accounted for 67% of the total polyphenol intake (about 1000 mg/d), followed by chocolate, apples, and vegetable oils (which accounted for 9%, 6%, and 3%, respectively, of the total polyphenol intake). Specifically, coffee was the main food source of hydroxycinnamic acids, thus enhancing the total phenolic acid intake, whereas tea was the main dietary source of flavonoids. Compared with other cohorts studied, the Polish HAPPIEE cohort consumed more coffee and tea. Eighty-three percent and 97% of the Polish cohort were consumers of coffee and tea (namely at least 1 cup/d), respectively: The mean intake of coffee and tea was 237 mL/d and 525 mL/d, respectively, and 79% of the population consumed >300 mL/d of tea (more than 2 cups/d). Mediterranean populations account for a very low consumption of tea and a relatively low intake of coffee. The French SU.VI.MAX cohort reported that 40% of women and 64% of men did not consume tea, with an average tea intake of 200 mL/d and only 34% having >300 mL/d [29]. As well, the Spanish PREDIMED cohort reported an average intake of about 50 mL/wk (0.4 ± 1.6 cups/wk) of tea and nearly 1 cup/d of coffee (6.5 ± 5.2 cups/wk, about 100 mL/d) [30]. Compared with our study, both aforementioned cohorts reported similar or slightly higher contribution of polyphenol intake from fruit and vegetables, and a significantly higher intake from alcohol beverages, due to a higher consumption of red wine [29], [30]. Another noteworthy difference between Mediterranean countries' polyphenol intake and the Polish HAPIEE cohort was the relevant contribution of seeds, vegetable oils, and cocoa products over the total amount of polyphenols (especially hydroxycinnamic acids). The Mediterranean cohorts (i.e., the SU.VI.MAX and the PREDIMED cohorts) reported that seeds and oils contributed to about 12 [13] and 32 mg/d [14] of polyphenols per person, whereas the Polish arm of the HAPIEE study accounted for about 72 mg/d, mostly depending on consumption of vegetable oil. As well, cocoa products accounted for ∼90 mg/d of polyphenols in the French cohort, ∼16 mg/d in the Spanish cohort, and 166 mg/d in the Polish HAPIEE cohort. These differences emphasize that the total and specific classes of polyphenols may vary according to country-specific food preferences. Despite non-Mediterranean countries sharing some common nutritional habits, such as high coffee and tea intake, the amount reported for the Finnish FINDIET 2002 cohort was much lower than that observed in the Polish cohort, showing a pooled coffee and tea intake of 409 ± 11 mL/d [21] (versus 762 mL/d in our study). Major contributors to polyphenol intake in the Finnish diet were tea, apples, and berries, similar to the Polish cohort, despite the fact that the average pooled intake of coffee and tea in the latter was nearly twice as high than the Finnish. No information was given about vegetable oils intake in the Finnish cohort, whereas total mean of cocoa products amounted to 667 mg/100 g. Thus, the differences in amounts of coffee, tea, vegetable oils, and seeds between Mediterranean and non-Mediterranean countries may justify the higher total amount of polyphenols consumed in the Polish cohort compared with the others, whereas type of fruits and vegetables may have influenced not only differences concerning polyphenols related to the aforementioned foods (i.e., flavanols, and hydroxycinnamic acids), but also regarding the other polyphenol classes.

Previous studies reported that polyphenol intake was influenced by sex, with men having higher absolute intakes of total polyphenols [13], [21], and specifically flavonoids [22], [31], than women due to higher consumption of coffee and wine. Although we also reported a slightly higher intake of polyphenols in men, the energy-adjusted intake revealed a higher energy density of polyphenols in women, suggesting that polyphenol intake in men is influenced by quantity of food, whereas in women it is by quality (i.e., higher consumption of tea). Significant differences in polyphenol intake were also due to age (higher intakes in younger participants), education, and physical activity. It may be speculated that a higher education (especially among younger participants) may mediate healthier lifestyles and eating behaviors, such as increased physical activity and consumption of fruit and vegetables, which are rich in polyphenols. On the contrary, this was not valid for drinking and smoking, both of which were associated with a higher intake of energy-adjusted polyphenol intake. It may be explained that alcoholic beverages (especially wine and beer) are contributors of polyphenols, and smokers were more likely to drink coffee, which was also one of the main contributors of polyphenol intake.

Recent research adopting the methodology of our study reported that polyphenol intake was significantly associated with a 46% reduction in risk for CVD when comparing the highest (1235 mg/d) versus the lowest (483 mg/d) quintile of intake [32]. The polyphenols with the strongest inverse associations were flavanols (263 mg/d), lignans (0.94 mg/d), and hydroxybenzoic acids (36.1 mg/d). We reported a similar intake of lignans but a general higher intake of both flavonoids and phenolic acids due to the higher consumption of tea and coffee, respectively. This data suggests that a significant part of our sample may benefit from the daily intake of polyphenols we reported. Further research would clarify whether the high consumption of such drinks and, consequently, increased intake of polyphenols in the Polish population could be translated to better health or protection against CVD.

Results of this study should be interpreted based on some limitations. First, differences between absolute intake of polyphenols in other studies are no doubt due to methodologic differences. Despite the source of data regarding polyphenols being the same among studies, the use of an FFQ may overestimate or rather give a more complete estimation of food consumption compared with 24-h recall. However, as discussed here, we consider this issue as marginal because most of the differences in polyphenol intake depended on coffee and tea consumption (which was actually much higher than in other countries), whereas the contribution of other foods rich in polyphenols (i.e., fruits and vegetables) was quite similar. Second, the FFQ used to collect dietary data referred to a limited amount of items and some foods rich in polyphenols were lacking (i.e., spices) or grouped (i.e., wine) leading to possible bias in the assessment of the total polyphenol intake. Third, due to the nature of the instrument, data may have been affected by recall bias and the low response rate (59%) may suggest that information could be representative of individuals with better health status [33].

## Conclusions

This study provided a complete description of the total polyphenol intake and main food contributors of dietary polyphenols in a Polish urban population. Together with the protective effects of vegetables and fruits against chronic diseases, the effects on health of other polyphenol-rich foods, such as tea, coffee, and cocoa products should be further investigated.
